# Culture-Dependent and Sequencing Methods Revealed the Absence of a Bacterial Community Residing in the Urine of Healthy Cats

**DOI:** 10.3389/fvets.2020.00438

**Published:** 2020-08-05

**Authors:** Andrea Balboni, Giovanni Franzo, Luca Bano, Stefano De Arcangeli, Alessia Rizzardi, Lorenza Urbani, Sofia Segatore, Federica Serafini, Francesco Dondi, Mara Battilani

**Affiliations:** ^1^Department of Veterinary Medical Sciences, Alma Mater Studiorum-University of Bologna, Ozzano Emilia, Italy; ^2^Department of Animal Medicine, Production and Health, University of Padua, Legnaro, Italy; ^3^Diagnostic and Microbiology Laboratory, Istituto Zooprofilattico Sperimentale delle Venezie, Villorba di Treviso, Italy

**Keywords:** bacterial community, cat, culture, microbiome, microbiota, sequencing, urinary tract, urine

## Abstract

A growing number of studies suggest that the lower urinary tract of humans and dogs can harbor a urinary microbiota. Nevertheless, a certain concern has developed that the microbiota reported could be due to unaccounted contamination, especially in low-biomass samples. The aim of this study was to investigate the bacterial community which populates the urine of healthy cats using two approaches: a culture-dependent approach which consisted of the expanded quantitative urine culture (EQUC) techniques capable of identifying live bacteria not growing in standard urine cultures, and a culture-independent approach which consisted of 16S ribosomal RNA next generation sequencing (16S rRNA NGS) capable of identifying bacterial DNA and exploring microbial diversity with high resolution. To avoid confounding factors of possible bacterial contamination, the urine was sampled using ultrasound-guided cystocentesis, and several sample controls and negative controls were analyzed. The urine sampled from the 10 cats included in the study showed no bacterial growth in the EQUC procedure. Although several reads were successfully originated using 16S rRNA NGS, a comparable pattern was observed between urine samples and the negative control, and no taxa were statistically accepted as non-contaminant. Taken together, the results obtained allowed stating that no viable bacteria were present in the urine of healthy cats without lower urinary tract disease and urinary tract infections, and that the bacterial DNA detected was of contaminant origin.

## Introduction

Until a few years ago, in human and veterinary medicine, the dogma that clinical urine specimens of asymptomatic patients are normally sterile was uniformly accepted as a result of the standard urine culture-negative status. Recently, the combined use of 16S ribosomal RNA gene next generation sequencing (16S rRNA NGS) and enhanced urine culture techniques allowed detecting bacterial members of the urinary microbiota previously unrecognized in standard urine cultures in humans (1, 2). Furthermore, urinary microbial diversities have been reported in patients of different ages and genders, or in healthy patients and those with urologic diseases, such as urinary incontinence or urolitiasis (3–5). Little knowledge regarding the bacterial communities which populate the urinary tract of domestic animals is available; a single study conducted on dogs revealed the presence of bacterial DNA in the bladder in the absence of a urinary tract infection (UTI) (6). To date, no investigation has been carried out in cats, although urinary tract diseases are among the most frequent in this species, and the etiology is often elusive (7). The characterization of the microbial community present in the urinary tract of healthy cats without urologic diseases can help to understand which bacteria are involved in the maintenance of urinary tract homeostasis and help the veterinary practitioner in the diagnosis and treatment of infections. In this prospective, the bacterial community which populates the urine of healthy cats without a history of clinical signs, and clinicopathological and microbiological findings associated with feline lower urinary tract disease (FLUTD) and UTI was investigated using both culture-dependent and culture-independent methods.

## Materials and Methods

### Study Design

This was a prospective study carried out in a Veterinary University Hospital in 2018–2019. Urine was sampled from ten healthy cats and the bacterial community was investigated using both culture-dependent and culture-independent methods to differentiate between live and dead bacteria or bacterial DNA fragments. The culture-dependent approach consisted of expanded quantitative urine culture (EQUC) techniques, partially modified when compared to those reported in previous studies (2, 8); the culture-independent approach consisted of 16S rRNA NGS to explore microbial diversity with a high depth of analysis (9).

To both avoid and to detect any contamination introduced by means of urine collection and processing which could be relevant due to the low microbial biomass environment of the bladder and urinary tract (10, 11), four approaches were adopted during the study: (1) ultrasound-guided cystocentesis was used for urine sampling, minimizing the introduction of extra-urinary bacterial contamination (7, 12); (2) culture-dependent and quantitative real-time PCR (qPCR) tests were carried out on skin swabs collected before and after skin disinfection to assess its effectiveness and evaluate the potential urine contamination by cutaneous microorganisms; (3) negative controls were carried out for each culture-independent test, and (4) a negative control was carried out for the 16S rRNA NGS, subjecting an aliquot of sterile phosphate-buffered saline (PBS) to each step of the DNA extraction procedure and bacterial 16S rRNA gene amplification, sequencing and bioinformatic analysis to identify bacterial DNA background contamination (13).

### Animals

Client-owned healthy cats were included in the study after owner consent if they had no history of clinical signs, and clinicopathological and microbiological findings indicative of FLUTD and UTI. To evaluate the healthy status of each cat, signalment, history and clinical data were recorded, and a complete blood count (CBC), serum chemical profile and urinalysis were carried out. Cats were excluded if bacteria grew in the standard urine culture (SUC). Animals who had undergone antibacterial and/or anti-inflammatory treatments over the previous 30 days were also excluded from the study.

Cystocentesis was performed using a 10 mL syringe after clipping the hair and surgical cleaning of the skin of the abdomen. A surgical scrub was carried out using 4% chlorhexidine gluconate (Neoxidina, Nuova Farmec) rinsed with isopropyl alcohol 70%. The procedure was repeated three times. Three skin swabs were sampled, one before and two after the surgical scrub, from each cat to evaluate potential urine contamination. One swab sampled before and one swab sampled after disinfection were independently suspended in 2 mL of BHI broth and used for the standard culture. One swab sampled post-disinfection was suspended in 200 μL of PBS and used for DNA extraction and 16S rRNA qPCR amplification.

For each cat included in the study, the urine sample was divided into four aliquots: (a) 5 mL for urinalysis, (b) 100 μL in 2 mL of brain heart infusion (BHI) broth (Difco) for the SUC, (c) 2 mL for the bacterial 16S rRNA gene sequencing, and (d) 2.5 mL for the EQUC.

The samples undergoing bacterial culture were conserved at 0–4°C and were processed within 8 h to minimize alterations in the bacterial concentration (14). The samples collected from each cat included in the study and the analyses they underwent are summarized in Supplementary Figure 1.

### Standard Culture

For each cat included in the study, a SUC was performed by inoculating 100 μL of urine in BHI broth, 10 μL on a 5% sheep blood agar plate (BAP) and 10 μL in MacConkey agar (MCA). The entire surface of the BAP and the MCA was streaked to obtain quantitative colony counts, and the plates and broths were incubated at 35°C in aerobic conditions and inspected after 24 and 48 h. In the absence of any growth after 24 h, 50 μL of the BHI broth were plated on a new BAP and new MCA and were incubated aerobically at 35°C for 24 and 48 h.

For each cat included in the study, two skin swabs in BHI broth, one sampled before and one after disinfection, were streaked on 2 plates of BAP, Eosin Methylene Blue (EMB) agar (Oxoid, Thermo Fisher Scientific, Waltham, MA, USA) and Baird Parker (BP) agar (Biokar Diagnostics, Allonne, France). One plate each of BAP, EMB, BHI and BP were incubated aerobically at 37°C. One plate of BAP was incubated at 37°C under anaerobic conditions. In the absence of growth in the plates streaked directly, the BHI was plated in agarized media (BAP, MCA, BP), incubated aerobically and inspected after 24 and 48 h.

The identification of the bacteria was carried out using the MALDI TOF MS (Biotyper Microflex LT, Bruker Daltonics, Billerica, MA, USA) with the MALDI Biotyper software package (version 3.0). Colonies with different morphology were sub-cultured to obtain an abundant and pure culture. Ten to 20 isolated colonies were picked up, suspended in 300 μl of sterile-filtered water (W3500, Sigma-Aldrich, St. Louis, MO, USA) and subjected to ethanol-formic acid protein extraction. Briefly, 900 μl of ethanol were added to the cell suspension, centrifuged at 20,000 rcf for 2 min, and the supernatant discharged. A second wash was performed, visible ethanol was discharged and the pellet was air-dried for at least 1 h. The pellet was then suspended in 20–50 μl (depending on pellet size) of formic acid-water solution (70/30; v/v) and vortexed. An equal volume of acetonitrile (Sigma-Aldrich, St. Louis, MO, USA) was added and, after thorough vortexing, the solution was centrifuged at 20,000 rcf for 2 min. Then, 1 μl of the supernatant was transferred to the MALDI target plate, allowed to dry at room temperature and overlaid with 1 μl of matrix. The matrix was obtained by dissolving 2.5 mg of α-cyano-4-hydroxy-cinnamic acid in an organic solvent composed of 500 μl of acetonitrile, 25 μl of trifluoroacetic acid (Bruker Daltonics, Billerica, MA, USA) and 475 μl of deionized water. The following settings were applied: positive linear mode; laser frequency 30 Hz; ion source 1 voltage, 19.98 kV, ion source 2 voltage, 17.79 kV; lens voltage, 7.0 kV and mass range, 1960 to 20,137 Da. The instrument was calibrated using the bacterial test strains (Bruker Daltonics, Billerica, MA, USA) according to the manufacturer's instructions. One spectrum was generated for each isolate from 240 laser shots in an automatic acquisition mode. The spectrum of each strain was matched with those contained in the reference database V.3.1.2.0 (Bruker Daltonics, Billerica, MA, USA). Identification reliability was scored, and an arbitrary value from “0” to “3” was assigned. As specified by the manufacturer, a score value lower than 1.7 indicated that the identification was not reliable; scores between 1.7 and 1.999 that the identification was reliable at the genus level; scores between 2.0 and 2.299 that the identification was reliable at the genus level and probable at the species level and scores higher than 2.3 indicated highly probable species identification. In case of missed identification (score value lower than 1.7), the bacterial species was indicated as “unidentified bacterial species.”

### Quantitative PCR

The DNA from post-disinfection skin swabs was extracted using the NucleoSpin Tissue Kit (Macherey-Nagel, Düren, Germany) according to the manufacturer's protocol. The presence of bacterial 16S rRNA gene DNA was evaluated in DNA extracts by SYBR Green qPCR carried out using the PowerUp SYBR Green Master Mix Kit (Life Technologies, Thermo Fisher Scientific, Waltham, MA, USA) and the StepOnePlus Real-Time PCR System (Life Technologies, Thermo Fisher Scientific, Waltham, MA, USA). Primers Bakt_341F (CCTACGGGNGGCWGCAG) and Bakt_805R (GACTACHVGGGTATCTAATCC) were used (15, 16). Extraction- and qPCR-negative controls were included to assess potential DNA contamination.

### Investigation of the Urine Bacterial Community Using the Culture-Dependent Approach

The urine sample (2.5 mL) underwent EQUC as previously described, with some modifications (2). The EQUC procedure use a variety of volumes of urine, culture media and incubation conditions to cultivate bacteria which do not grow under standard culturing conditions (8, 11). For EQUC, 100 μL of the urine samples were plated in (i) BAP incubated at 35°C in a microaerophilic chamber (Bactron 300, Sheldon Manufacturing, Cornelius, OR, USA) with an atmosphere composed of 5% O_2_, 10% CO_2_ and 85% N_2_; (ii) chocolate colistin and nalidixic acid (CNA) agar (Biolife Italiana, Mascia Brunelli, Milan, Italy) incubated in microaerophilic conditions; (iii) Brucella Agar (BA) (Becton Dickinson, Franklin Lakes, NJ, USA), supplemented with hemin (5 μg/mL), vitamin K1 (1 μg/mL), laked sheep blood (5% v/v) and L-cysteine (0.4 g/L) incubated for 48 h at 35°C in an anaerobic cabinet (Bactron IV, Sheldon Manufacturing, Cornelius, OR, USA) with an atmosphere composed of 5% H_2_, 10% CO_2_ and 85% N_2_ and (iv) two BAPs incubated under aerobic conditions, one at 35°C and one at 30°C. In addition, 0.5 mL of each sample was added to 4.5 mL of Veterinary Fastidious broth (17) which was incubated under anaerobic conditions at 35°C for 48 h and subsequently plated on a BA and a BAP, incubated at 35°C under anaerobic and microaerophilic conditions, respectively, and both inspected at 24 and 48 h (2, 17). The bacteria were identified using MALDI TOF MS with the instrument setting reported above.

### Investigation of the Urine Bacterial Community Using the Culture-Independent Approach

The DNA from 2 mL of urine sample and 2 mL of PBS as a negative control to 16S rRNA gene sequencing (NCS) was extracted using the QIAamp DNA Microbiome Kit (Qiagen, Hilden, Germany) according to the manufacturer's protocol. Before extraction, the urine sample was pelleted (2,600 rcf for 10 min) and the pellet was resuspended in 500 μL of PBS. The DNA extracted was eluted in 50 μL of Buffer AVE and stored at −20°C. Total DNA was quantified using a Qubit fluorometer (Life Technologies, Thermo Fisher Scientific, Waltham, MA, USA).

A DNA fragment containing the hypervariable regions 3 and 4 (V3-V4) of the bacterial 16S rRNA gene was amplified from each urine and NCS sample and was sequenced using a custom protocol developed for the Illumina MiSeq sequencer platform (San Diego, CA, USA) by BioFab Research (Rome, Italy).

The bacterial 16S rRNA gene amplicon library was generated by the PCR amplification of the V3-V4 hypervariable regions carried out with KAPA HiFi HotStart DNA Polymerase (Roche Sequencing Solutions, Pleasanton, CA, USA) and the universal primers Bakt_341F and Bakt_805R complemented with Illumina adapter sequences (15, 16). Each sample was amplified with 25 PCR cycles under the following conditions: 95°C for 30 s, 55°C for 30 s and 72°C for 30 s. A negative control in the form of a PCR-amplified ultrapure water sample was included in the reaction.

The amplicons were analyzed by gel electrophoresis and were purified; the DNA concentration of the eluted product was determined using a Qubit fluorometer (Life Technologies, Thermo Fisher Scientific, Waltham, MA, USA). The DNA samples were sequenced with an Illumina MiSeq sequencer platform using a paired-end 2 × 300-bp reagent cartridge according to the manufacturer's instructions (MiSeq Reagent Kit v3, Illumina, San Diego, CA, USA). The PCR amplification and sequencing of all the urine and the NCS samples were carried out simultaneously in the same run to avoid introducing variability between different reactions.

The raw sequences were processed using the open-source program DADA2 pipeline (18)^1^. In particular, the reads were trimmed to remove primer sequences; reads with ambiguous, poor quality bases (phred quality score lower than 20) and more than two expected errors were discarded. The reads obtained were dereplicated and denoised using the DADA2 algorithm after estimating the error rates from the data, by alternating between sample inference and error rate estimation until convergence. The paired reads were then merged, and the chimeras identified and removed. Finally, the taxonomy was assigned to the sequence variants using the naive Bayesian classifier method implemented in DADA2. To this purpose, the Silva taxonomic training data formatted for DADA2 [version 132; (19)] was used as a reference. Different metrics of alpha diversity (“Chao1,” “ACE,” and “Shannon”) were calculated using phyloseq R Packages. The presence of contaminant reads was assessed by comparing the taxa prevalence in the urine samples with the NCS sample, using the statistical approach implemented in the *is Not Contaminant* function of the *decontam* package in R, which is recommended for low biomass samples, such as urine (20). A conservative *P* < 0.1 threshold was selected for removing the contaminant reads. Sample processing and graphics were created using the *phyloseq and microbiome* libraries implemented in R.

## Results

### Study Population

Ten healthy cats were included in the study. No bacteria grew in the SUC carried out for each cat. Four out of the 10 cats included in the study were castrated males, and 6/10 were spayed females. The median age was 5 years (range 2–14 years). Eight were domestic shorthaired cats, one was a Devon rex and one was a Persian. Half of the cats (5/10) were indoor/outdoor and half were exclusively indoor. The clinicopathological findings of the cats included in the study are reported in the Supplementary Table 1.

### Evaluation of the Effectiveness of Skin Disinfection Performed Before the Ultrasound-Guided Cystocentesis

A standard culture carried out on pre-disinfection skin swabs showed the growth of several bacteria species belonging to Proteobacteria and Firmicutes phyla (*Bacillus, Paenibacillus, Streptococcus, Acinetobacter, Staphylococcus, Paracoccus* and *Pasteurella* genera). No bacteria grew in the standard culture, and no bacterial 16S rRNA gene DNA was revealed by a qPCR carried out on post-disinfection skin swab samples.

### Investigation of the Urine Bacterial Community Using the Culture-Dependent and Culture-Independent Approaches

No bacteria grew with the partially modified EQUC techniques carried out on the urine samples taken by cystocentesis from each cat included in the study.

**Figure 1:**
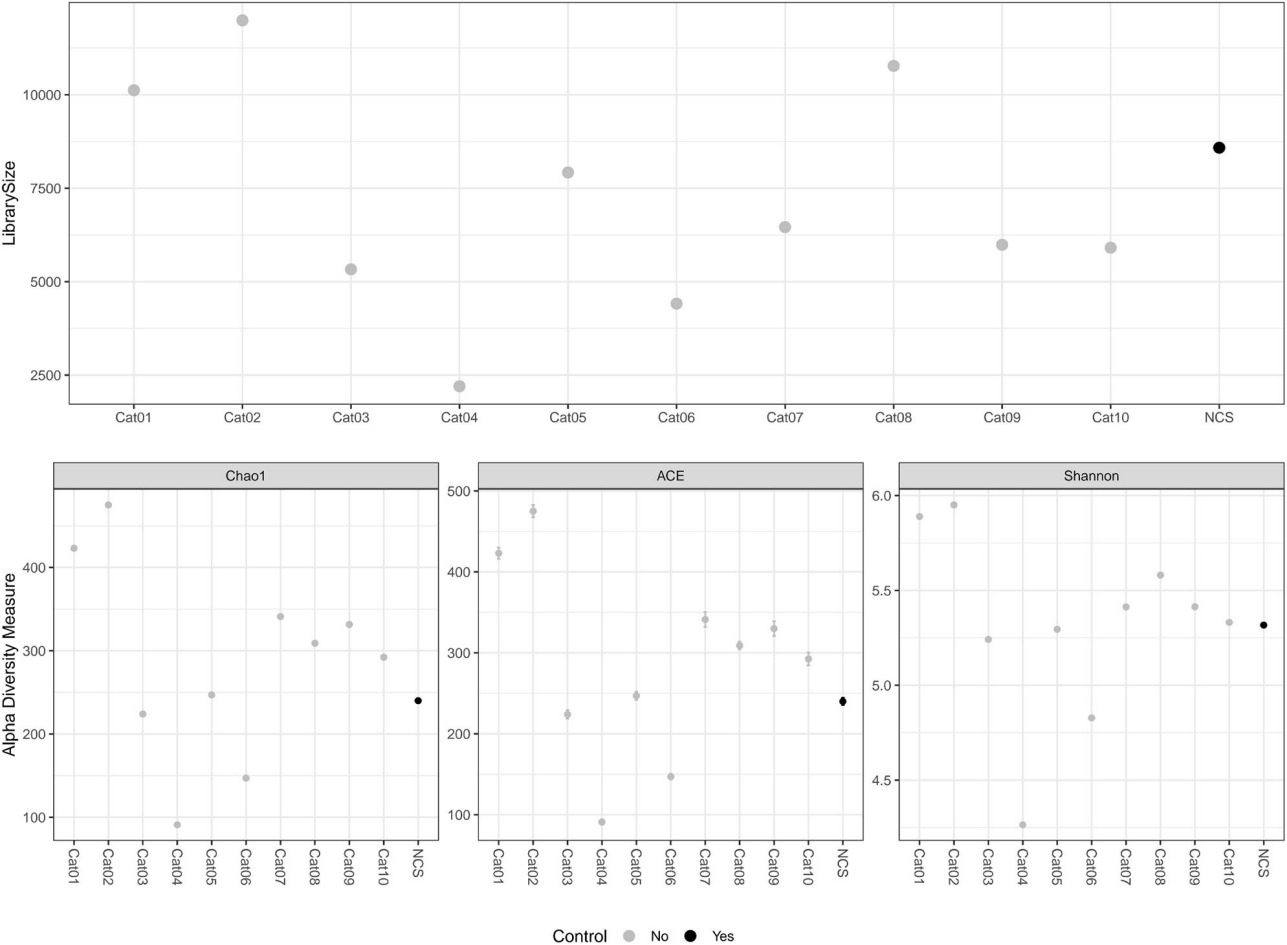
Library size and alpha diversity evaluation. The library size of the different samples included in the study are reported in the top graph. The alpha diversity measurements are reported in the graphs below. Different metrics of alpha diversity (“Chao1”, “ACE,” and “Shannon”) were calculated using the phyloseq R Packages. NCS, negative control to 16S rRNA gene sequencing.

The DNA concentration in the eluted extracts obtained from the urine and from the NCS samples was lower than the limit of detection of the Qubit fluorometer (0.5 ng/mL) while the DNA concentration of the libraries obtained by the amplification of the hypervariable regions V3-V4 of the bacterial 16S rRNA gene are shown in Table 1. The total number and length of the reads obtained by Illumina MiSeq sequencing from each DNA sample, and the number of reads after filtering, denoising, merging and chimera removal are reported in Table 1. The final number of merged reads ranged from 2,202 to 11,991 (median 6,460). The final number of reads in the NCS (8,584) was in the upper part of this range (Figure 1). Likewise, the evaluation of several alpha diversity indices revealed similar values among the samples, including the negative control.

**Table 1:** Summary of bacterial 16S rRNA gene amplicon library features before and after processing.

**Sample**	Library concentration (ng/μL)^*^	**Reads obtained (mean nucleotide length)**	**Reads filtered**	**Forward reads denoised**	**Reverse reads denoised**	**Reads merged**	**Chimera removal**
Cat01	3.3	64,128 (247)	22,401	21,919	21,478	20,813	10,122
Cat02	2.3	47,933 (268)	19,548	19,095	19,055	16,592	11,991
Cat03	1.5	36,270 (251)	13,460	13,392	13,139	13,039	5,330
Cat04	5.8	117,967 (72)	5,715	5,491	5,517	4,993	2,202
Cat05	1.2	45,722 (208)	13,712	13,336	13,397	12,594	7,921
Cat06	1.3	39,628 (296)	8,929	8,750	8,706	8,429	4,413
Cat07	0.8	29,501 (284)	13,615	12,593	12,245	8,525	6,460
Cat08	2.1	42,203 (252)	17,558	16,981	17,132	15,259	10,773
Cat09	1.1	27,962 (293)	12,814	11,927	11,742	8,464	5,986
Cat10	0.9	21,982 (294)	10,254	9,765	9,521	7,400	5,909
NCS	2.4	49,022 (202)	13,725	13,079	13,318	12,433	8,584

*Total number and length of the reads obtained from each DNA sample, and the number of reads after filtering, denoising, merging and chimera removal are reported. The raw sequences were processed using the open-source program DADA2 pipeline (18)^1^*.

* *Concentration of the bacterial 16S rRNA gene amplicon libraries determined using the Qubit fluorometer (Thermo Fisher Scientific, Life Technologies). NCS, negative control to 16S rRNA gene sequencing*.

**Figure 2:**
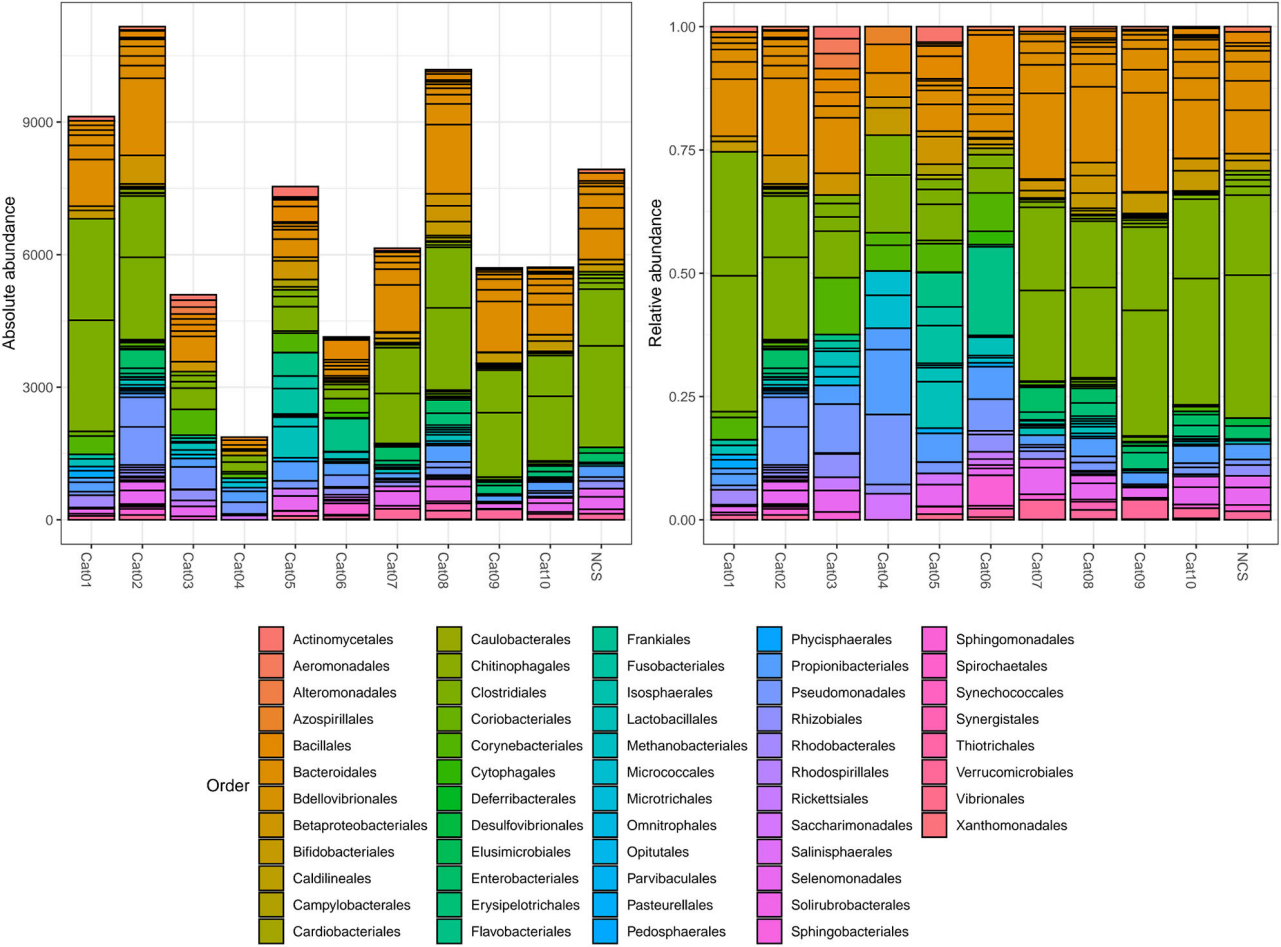
Within subject absolute and relative abundance of the taxa present in different orders (color coded). The taxonomy was assigned to the sequence variants using the naive Bayesian classifier method implemented in DADA2 (18)^1^. The Silva taxonomic training data formatted for DADA2 [version 132; (19)] was used as a reference. NCS, negative control to 16S rRNA gene sequencing.

**Figure 3:**
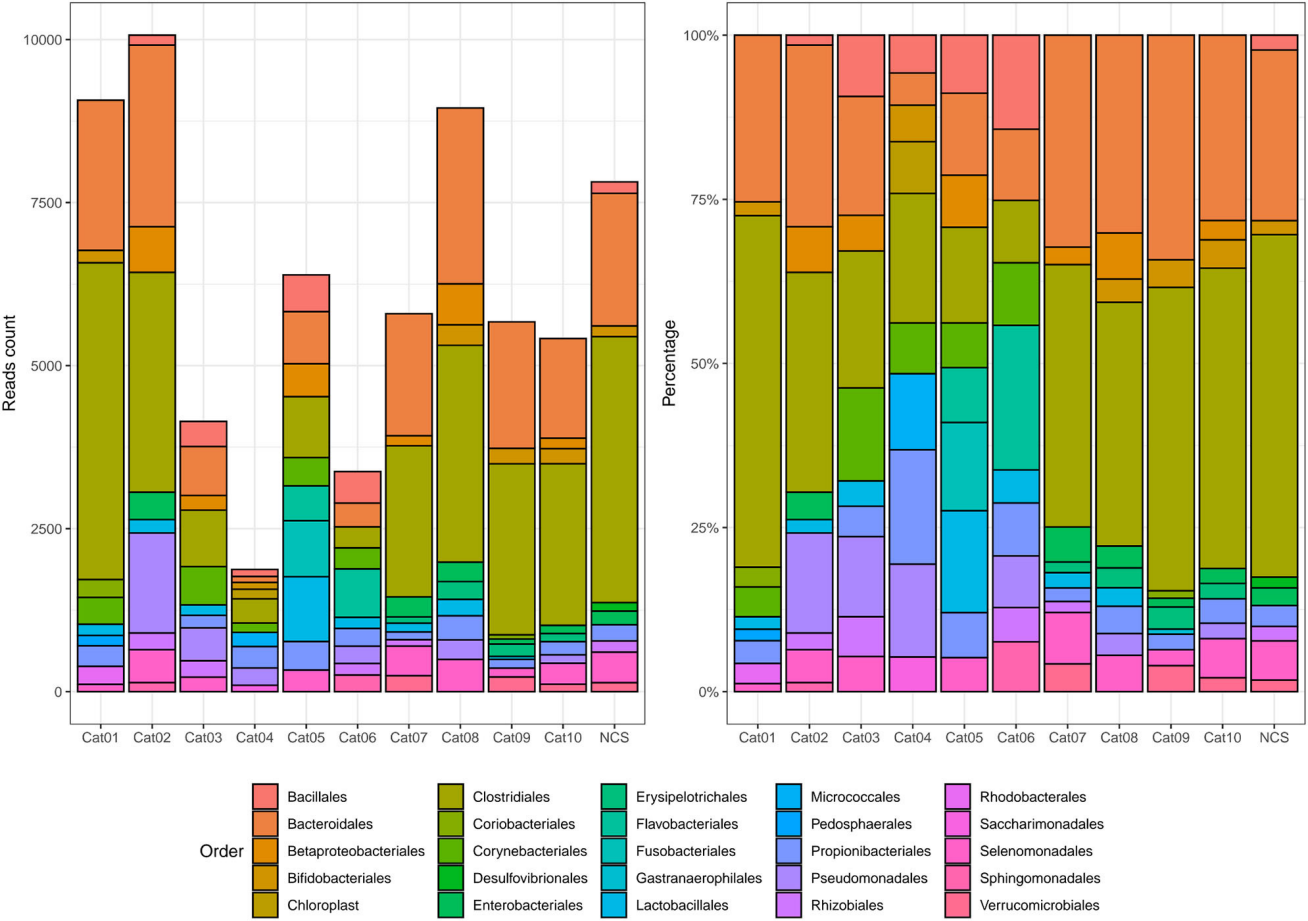
Absolute and relative abundance of the 10 most prevalent taxa present in each subject (color coded). The taxonomy was assigned to the sequence variants using the naive Bayesian classifier method implemented in DADA2 (18)^1^. The Silva taxonomic training data formatted for DADA2 [version 132; (19)] was used as a reference. NCS, negative control to 16S rRNA gene sequencing.

Bacteria belonging to several orders could be identified in the urine and in the NCS DNA samples (Figure 2). However, if the 10 most abundant taxa for each sample were selected, all the urine and the NCS samples demonstrated a similar composition, although a certain difference in the relative abundance of bacterial orders was observed in some cats (i.e., cats 3, 4, 5, and 6) (Figure 3 and Supplementary Table 2). Bacteria belonging to a total of 25 orders were predominantly present, but only 10 orders were present in more than half (6/11) of the samples analyzed: Bacillales, Bacteroidales, Betaproteobacteriales, Bifidobacteriales, Clostridiales, Enterobacteriales, Lactobacillales, Propionibacteriales, Pseudomonadales, and Selenomonadales. Of those, Bacteroidales, Clostridiales, Propionibacteriales and Selenomonadales were present in the largest number of the urine and the NCS samples (at least 9 out of 11), with Bacteroidales and Clostridiales being widely present in all samples and having the highest number of reads (Supplementary Table 2). All the other taxa were present in the majority of the samples at a lower and variable frequency. However, the contaminant read analysis carried out with *decontam* evidenced that no read could be confidently considered as non-contaminant. Beta diversity (bacterial community composition) was not calculated in this study since the reads obtained from the urine samples of the 10 cats and from the NCS did not pass the non-contamination test, thus making the potentially obtained results not reliable.

## Discussion

The prevailing assumption that clinical urine specimens of asymptomatic patients can be considered sterile was recently questioned in human medicine following emerging evidence claiming that the lower urinary tract could have urinary microbiota (1–3, 5, 8). With the exception of a study reporting the presence of bacterial DNA in the urine of healthy dogs (6), to date, no data on the bacterial communities that populate the urinary tract of domestic animals are available. However, while an increasing number of studies have highlighted the presence of resident microbiota in different body sites, and its role in physiological and pathological processes, mounting awareness has also increased regarding false positive results due to contamination (13). Due to the extreme sensibility of NGS technology, minimal DNA contamination originating from the sampling site, environment, sampling tools, personnel and used reagents can lead to inaccurate data interpretation, especially in low-biomass samples (10, 11).

In this study, the bacterial community which populates the urine of healthy cats was investigated using two approaches: a culture-dependent approach which consisted of the EQUC techniques capable of identifying live bacteria not growing in a standard urine culture (2, 8), and a culture-independent approach which consisted of 16S rRNA NGS capable of identifying bacterial DNA and exploring microbial diversity with high resolution (9). To avoid the confounding factors of possible bacterial contamination introduced through urine collection and processing, the urine was directly sampled from the feline bladders using ultrasound-guided cystocentesis. Furthermore, several sample controls and negative controls were taken and analyzed using both culture-dependent and culture-independent methods. In particular, skin swabs collected before and after the disinfection performed during the cystocentesis were tested to reveal potential urine contamination by skin microorganisms and to check the efficacy of the cleaning procedures. In addition, a negative control for the 16S rRNA NGS (NCS) was analyzed in order to identify the bacterial DNA background likely introduced by reagents and materials.

The tests carried out on the control samples confirmed the effectiveness of the skin disinfection performed before ultrasound-guided cystocentesis; therefore, no viable bacteria or bacterial DNA was introduced as a contaminant in the urine sampled. The bacterial species identified by standard culture in the skin of sampled cats before the disinfection were included among those previously reported in the skin of healthy cats by Older and collaborators (21).

The urine sampled from the 10 cats included in the study had no bacterial growth in the EQUC procedure, confirming the SUC results and suggesting that viable bacteria were not present in the urine of healthy cats. However, it must be considered that some bacterial species grow inside the cells (intracellular bacteria), others grow on cell cultures and still others are not at all cultivable (22).

The presence of bacterial DNA in the urine of the cats sampled was investigated using amplification and sequencing of the hypervariable regions V3-V4 of the bacterial 16S rRNA gene. Although the concentration of DNA in all the urine and the NCS samples was lower than the Qubit limit of detection, the libraries obtained were sequenced, originating a low number of reads. Several reads were successfully classified at least at the genus level and a comparable pattern could be observed between the urine and the NCS samples. Although some cats displayed a different profile in terms of order abundance, probably due to the variable efficiency of the bacterial 16S rRNA gene amplification in low microbial biomass samples, from a qualitative perspective all animals and the NCS were largely comparable. Accordingly, when the presence of contaminant sequences was statistically assessed, no reads could be confidently classified as non-contaminant. In support of the contaminant origin of the bacterial DNA detected, several bacterial orders, grouping the most abundant taxa herein identified, were reported as contaminant in sequenced negative “blank” controls by Salter and collaborators (13). Therefore, these bacterial reads could likely originate from the reagents and extraction kits used to process the samples in the present study. Additionally, other sources of contamination can not be excluded, and, despite all the efforts paid to limit them, may have involved laboratory materials and personnel, justifying the presence of some previously unreported contaminants.

Another, non-conflicting hypothesis which could explain the presence of the bacterial reads obtained by 16S rRNA NGS in feline urine is the ultrafiltration of bacterial DNA. Ever-increasing evidence is accumulating regarding the presence of a “healthy” blood microbiome in human beings (23). Blood-borne bacteria could exploit a viable ecological niche or simply be transient residents in the blood after translocation from other body-sites, such as damaged skin, and oral or intestinal mucosa (24, 25). Furthermore, even an intact intestinal epithelial barrier could allow the entry of intestinal bacteria into the circulatory system mediated by cells of the immune system or by related cells, such as intestinal M-cells (26–30). A similar phenomenon could also occur in animals, such as cats. Although the intact bacterial genome cannot pass a functional renal filter, a fragmented region including the 16S could pass to the glomerular filtrate (31). This scenario could explain the differences among subjects and with the NCS. However, contradictory evidence is present regarding this topic, and additional studies should be carried out to evaluate the plausibility of this hypothesis (32).

While the minor difference in the taxa detected between the urine and the NCS samples could lead to hypothesizing the presence of a certain resident microbiome, it must be stressed that the comparison of read counts alone could lead to misleading result interpretation. In fact, relative abundance thresholds can remove rare features (i.e., taxa) that are truly present in the sample, while not removing abundant contaminants. Another common approach is the removal of sequences which appear in the negative controls. However, cross-contamination between samples often causes abundant true sequences to be detected in negative controls. For these reasons the Authors considered a contaminant classification based on a probabilistic approach to be more reliable. According to these criteria, no taxa could confidently be accepted as actually present in the urine samples.

Furthermore, almost all the studies carried out on humans have reported *Lactobacillus* as the most abundant genera detected by the EQUC protocol and/or 16S rRNA sequencing in the urine of both healthy women and men (1–4, 33). *Lactobacillus* was commonly found in women's vaginal microbiome, but it was assumed that it was probably not a urinary tract contaminant due to the urine collection route used in those studies. On the contrary, in the present study, the taxa belonging to this genus were identified only inconsistently (7 out 10 subjects) and in marginal quantities (<1%) by 16S rRNA NGS. This discrepancy could be the consequence of a different urinary bacterial population between the two species, but also additional evidence to support the lack of a urinary microbiota in healthy cats.

Taken together and according to a caution principle, the results obtained, from both the culture-dependent and the culture-independent approaches, along with stringent inclusion criteria, the urine sampling technique used and the control systems adopted both to avoid and to detect any contamination, allowed stating that no viable bacteria were present in the urine of healthy cats without urinary tract disease and that the bacterial DNA detected was of contaminant origin.

The low number of healthy cats included in the analysis represented the main limitation of the present study. Nevertheless, comparable results were obtained in all the subjects tested, reducing the possibility of having been misled in their analysis due to sampling errors or between-subject variability. A further limitation of the study is the absence of intact animals in the analyzed population, as a consequence of the inclusion criteria adopted, based on the health status of cats and not on sex. Future studies with a larger and well-characterized study population will be required to confirm the present results and eventually evaluate the differences between subgroups divided by sex or age.

In conclusion, the results herein reported suggested that healthy cat urine is sterile and once more stressed the need to carefully account for the presence of potential contaminants despite all the efforts paid to preventing them in the sample processing steps. This study also evidenced that the adoption of sampling methods to ensure sterility, and of negative control samples to reveal bacterial 16S rRNA gene background contamination are of fundamental importance in correctly evaluating the bacterial communities. However, the possibility that diverse bacteria can exist within the cat urinary tract, having important implications for urinary tract diseases as the intestinal microbial community structure has been shown to influence susceptibility to infection (34), warrants additional extensive studies to investigate the microbial community present in the urinary tract of cats with urologic diseases associated or not with UTI.

## Data Availability Statement

The FASTQ reads generated in this study can be found in the National Center for Biotechnology Information and registered under SRA (https://trace.ncbi.nlm.nih.gov/Traces/sra) accession nos. SRR11434303-SRR11434313 [BioProject (https://www.ncbi.nlm.nih.gov/bioproject) accession no. PRJNA613836].

## Ethics Statement

The animal study was reviewed and approved by Animal Welfare Committee (COBA) of the Alma Mater Studiorum - University of Bologna (Bologna DL 26/2014, Project 862). Written informed consent was obtained from the owners for the participation of their animals in this study.

## Author Contributions

AB, GF, and LB designed the study, analyzed data, co-wrote, and edited the manuscript. SD, AR, LU, SS, and FS assisted with study design, collected, and analyzed data. FD and MB supervised and edited the manuscript. All authors contributed to read and approved the final manuscript.

## Conflict of Interest

The authors declare that the research was conducted in the absence of any commercial or financial relationships that could be construed as a potential conflict of interest.

## Footnotes

^1^DADA2 pipeline. https://github.com/benjjneb/dada2 (accessed February 01, 2020).

## Abbreviations

16S rRNA NGS: 16S ribosomal RNA gene next generation sequencing
BA: Brucella Agar
BAP: blood agar plate
BHI: brain heart infusion
BP: Baird Parker
CBC: complete blood count
CNA: colistin and nalidixic acid
EMB: Eosin Methylene Blue
EQUC: expanded quantitative urine culture
FLUTD: feline lower urinary tract disease
K3EDTA: ethylene diamine tetra-acetic acid
MCA: MacConkey agar
NCS: negative control to 16S rRNA gene sequencing
PBS: phosphate-buffered saline
qPCR: quantitative real-time PCR
rcf: relative centrifugal force
SC: standard culture
SUC: Standard urine culture
UTI: urinary tract infection
V3-V4: hypervariable regions 3 and 4.
