# Supplementary material for: Culture-Dependent and Sequencing Methods Revealed the Absence of a Bacterial Community Residing in the Urine of Healthy Cats
Source: Front Vet Sci. 2020 Aug 5;7:438. doi: 10.3389/fvets.2020.00438 (PMC7419613; doi:10.3389/fvets.2020.00438)
Supplement: Supplementary file 1 [file Data_Sheet_1.docx]

Supplementary Material

**Supplementary Table 1.** Clinicopathological findings of the cats included in the study

|  |  | Cats included in the study | Reference interval |
| --- | --- | --- | --- |
| Haematology | |  |  |
|  | RBCs (10^3^ cells/mm^3^) | 9270 (7550 – 10870) | 7000 – 11000 |
|  | WBCs (cells/mm^3^) | 7530 (6650 – 8160) | 4800 – 14930 |
|  | Hb (gr%) | 13.7 (11 – 15.9) | 10 – 16 |
|  | Hct (%) | 40.8 (35.4 – 45.5) | 32 – 48 |
|  | MCV (fL) | 42.8 (41.1 – 49.1) | 36 – 55 |
|  | MCH (pg) | 14.1 (13 – 15.4) | 12.3 – 16.2 |
|  | MCHC (g%) | 33.5 (31.8 – 35.1) | 31 – 36 |
|  | MPV (fL) | 15.8 (12.3 – 26) | 8 – 26 |
|  | RDW (%) | 14.3 (13 – 16.1) | 13.17 – 17 |
|  | Platelets (cells/mm^3^) | 274000 (204000 – 356000) | 150000 – 500000 |
|  | Reticulocytes (cells/mm^3^) | 34150 (9200 – 66100) | 0 – 80000 |
|  | Neutrophils (cells/mm^3^) | 3575 (2650 – 6860) | 1600 – 10000 |
|  | Monocytes (cells/mm^3^) | 130 (90 – 210) | 0 – 650 |
|  | Lymphocytes (cells/mm^3^) | 3220 (390 – 4440) | 900 – 5600 |
|  | Basophils (cells/mm^3^) | 10 (10 – 20) | 0 – 60 |
|  | Eosinophil (cells/mm^3^) | 550 (140 – 780) | 60 – 1470 |
| Serum Chemistry | |  |  |
|  | ALT (U/L) | 51 (30 – 72) | 20 – 72 |
|  | AST (U/L) | 31 (17 – 37) | 9 – 40 |
|  | ALP (U/L) | 31 (20 – 53) | 0 – 140 |
|  | GGT (U/L) | 0.1 (0.1 – 2.3) | 0 – 4 |
|  | Total Bilirubin (mg/dL) | 0.17 (0.07 – 0.21) | 0 – 0.3 |
|  | Cholesterol (mg/dL) | 138 (121 – 197) | 64 – 229 |
|  | Glucose (mg/dL) | 81 (70 – 113) | 63 – 148 |
|  | Albumin (g/dL) | 3.32 (2.99 – 3.69) | 2.6 – 4 |
|  | Total Protein (g/dL) | 7.87 (6.61 – 8.74) | 6.5 – 8.8 |
|  | A:G (g:g) | 0.8 (0.55 – 1.04) | 0.52 – 1.19 |
|  | Creatinine (mg/dL) | 1.34 (0.99 – 1.6) | 0.8 – 1.8 |
|  | Urea (mg/dL) | 50 (36 – 56) | 30 – 65 |
|  | Phosphate (mg/dL) | 4.39 (3.46 – 5.13) | 2.5 – 6.2 |
|  | Potassium (mEq/L) | 4.25 (3.7 – 5.1) | 3.4 – 5.1 |
|  | Sodium (mEq/L) | 153 (151 – 155) | 145 – 155 |
|  | Chloride (mEq/L) | 118 (115 – 121) | 110 – 123 |
|  | Magnesium (mg/dL) | 2.28 (2.03 – 2.71) | 1.9 – 2.8 |
|  | Total Calcium (mg/dL) | 9.5 (9.3 – 10.5) | 8.5 – 10.5 |
| Urinalysis and chemistry | |  |  |
|  | USG | 1054 (1044 – 1085) | >1040 |
|  | Glycose (mg/dL) | Negative | Negative |
|  | Urine creatinine (mg/dL) | 315 (184 – 386) | – |
|  | Urine protein (mg/dL) | 60 (38 – 73) | – |
|  | UPC (mg:mg) | 0.20 (0.10 – 0.31) | <0.4 |
|  | microscopic sediment examination | Negative |  |

Data are reported as median and range (min – max). Blood samples were collected by standard venipuncture using a blood vacuum collection system (Vacutest Kima, Arzergrande, Italy), and concomitant urine samples were obtained using ultrasound-guided cystocentesis. For each cat, a K3 ethylene diamine tetra-acetic acid (K3EDTA) blood sample was used for the CBC, and the serum sample underwent a chemical profile. The complete blood count (CBC) was carried out using an automated haematology analyzer (ADVIA 2120; Siemens Healthcare Diagnostics). Haematological evaluation was completed with a microscopic blood smear examination using May-Grünwald Giemsa staining. Serum chemistry profile was determined as reported previously (L. Urbani, A. Tirolo, D. Salvatore, M. Tumbarello, S. Segatore, M. Battilani, A. Balboni, F. Dondi, Serological, molecular and clinicopathological findings associated with *Leishmania infantum* infection in cats in Northern Italy, J. Feline. Med. Surg. (2020). https://doi.org/10.1177/1098612X19895067). Urinalysis included urine specific gravity, dipstick (Combur10TestUX; Roche), microscopic sediment examination and the urine protein:creatinine ratio (UPC). Serum and urine chemical analysis were carried out using an automated analyzer (AU480; Beckman Coulter-Olympus).

A:G, albumin to globulin ratio; ALP, alkaline phosphatase level; ALT, alanine aminotransferase; AST, aspartate aminotransferase; GGT, gamma(γ)-glutamyl transferase; Hb, hemoglobin; Hct, hematocrit value; MCH, mean corpuscular haemoglobin; MCHC, mean corpuscular haemoglobin concentration; MCV, mean corpuscular volume; MPV, mean platelet volume; RBCs, red blood cells; RDW, red cell distribution width; UPC, urine protein:creatinine ratio; USG, urine specific gravity; WBCs, white blood cells.

**Supplementary Table 2.** Absolute and relative abundance of the 10 most prevalent taxa present in each subject.

|  | **Cat01** | **Cat02** | **Cat03** | **Cat04** | **Cat05** | **Cat06** | **Cat07** | **Cat08** | **Cat09** | **Cat10** | **NCS** | **median [range] ^b^** |
| --- | --- | --- | --- | --- | --- | --- | --- | --- | --- | --- | --- | --- |
| Bacillales |  | 152 (1.3) | 386 (7.2) | 108 (4.9) | 564 (7.1) | 483 (10.9) |  |  |  |  | 177 (2.1) | 6.0 [1.3-10.9] |
| Bacteroidales | 2303 (22.7) | 2785 (23.2) | 751 (14.1) | 92 (4.2) | 799 (10.1) | 366 (8.3) | 1870 (28.9) | 2697 (25) | 1940 (32.4) | 1528 (25.9) | 2032 (23.7) | 23.2 [4.2-32.4] |
| Betaproteobacteriales |  | 699 (5.8) | 225 (4.2) |  | 506 (6.4) |  | 155 (2.4) | 628 (5.8) |  | 160 (2.7) |  | 5.0 [2.4-6.4] |
| Bifidobacteriales | 190 (1.9) |  |  | 104 (4.7) |  |  |  | 316 (2.9) | 238 (4) | 233 (3.9) | 165 (1.9) | 3.4 [1.9-4.7] |
| Chloroplast |  |  |  | 148 (6.7) |  |  |  |  |  |  |  | 6.7 [6.7-6.7] |
| Clostridiales | 4858 (48) | 3372 (28.1) | 865 (16.2) | 370 (16.8) | 933 (11.8) | 321 (7.3) | 2317 (35.9) | 3325 (30.9) | 2621 (43.8) | 2478 (41.9) | 4079 (47.5) | 30.9 [7.3-48] |
| Coriobacteriales | 272 (2.7) |  |  |  |  |  |  |  | 65 (1.1) |  |  | 1.9 [1.1-2.7] |
| Corynebacteriales | 413 (4.1) |  | 587 (11) | 145 (6.6) | 435 (5.5) | 322 (7.3) |  |  |  |  |  | 6.6 [4.1-11] |
| Desulfovibrionales |  |  |  |  |  |  |  |  |  |  | 129 (1.5) | 1.5 [1.5-1.5] |
| Enterobacteriales |  | 421 (3.5) |  |  |  |  | 308 (4.8) | 298 (2.8) | 76 (1.3) | 124 (2.1) | 210 (2.4) | 2.6 [1.3-4.8] |
| Erysipelotrichales |  |  |  |  |  |  | 94 (1.4) | 270 (2.5) | 190 (3.2) | 126 (2.1) |  | 2.3 [1.4-2.3] |
| Flavobacteriales |  |  |  |  | 533 (6.7) | 743 (16.8) |  |  |  |  |  | 11.8 [6.7-16.8] |
| Fusobacteriales |  |  |  |  | 860 (10.9) |  |  |  |  |  |  | 10.9 [10.9-10.9] |
| Gastranaerophilales |  |  |  |  |  |  |  |  | 45 (0.7) |  |  | 0.7 [0.7-0.7] |
| Lactobacillales | 172 (1.7) | 204 (1.7) | 160 (3) |  | 992 (12.5) | 170 (3.8) | 135 (2.1) | 254 (2.4) |  |  |  | 2.4 [1.7-12.5] |
| Micrococcales |  |  |  | 217 (9.8) |  |  |  |  |  |  |  | 9.8 [9.8-9.8] |
| Pedosphaerales | 158 (1.6) |  |  |  |  |  |  |  |  |  |  | 1.6 [1.6-1.6] |
| Propionibacteriales | 314 (3.1) |  | 192 (3.6) | 327 (14.8) | 438 (5.5) | 272 (6.2) | 120 (1.9) | 370 (3.4) | 134 (2.2) | 201 (3.4) | 249 (2.9) | 3.4 [1.9-14.8] |
| Pseudomonadales |  | 1535 (12.8) | 506 (9.5) | 265 (12) |  | 266 (6) |  | 298 (2.8) |  | 128 (2.2) |  | 7.8 [2.2-12.8] |
| Rhizobiales |  | 256 (2.1) | 251 (4.7) |  |  | 176 (4) | 97 (1.5) |  |  |  | 172 (2) | 2.1 [1.5-4.7] |
| Rhodobacterales | 277 (2.7) |  |  |  |  |  |  |  |  |  |  | 2.7 [2.7-2.7] |
| Saccharimonadales |  |  |  | 99 (4.5) |  |  |  |  |  |  |  | 4.5 [4.5-4.5] |
| Selenomonadales | 113 (1.1) | 505 (4.2) | 222 (4.2) |  | 332 (4.2) |  | 454 (7) | 495 (4.6) | 137 (2.3) | 322 (5.4) | 468 (5.4) | 4.2 [1.1-7] |
| Sphingomonadales |  | 138 (1.1) |  |  |  | 256 (5.8) |  |  |  |  |  | 3.5 [1.1-5.8] |
| Verrucomicrobiales |  |  |  |  |  |  | 245 (3.8) |  | 225 (3.8) | 115 (1.9) | 137 (1.6) | 2.9 [1.6-3.8] |
| Top 10 taxa | 9070 (89.6) | 10067 (83.9) | 4145 (77.8) | 1875 (85.1) | 6392 (80.7) | 3375 (76.5) | 5795 (89.7) | 8951 (83.1) | 5671 (94.7) | 5415 (91.6) | 7818 (91.1) | 85.1 [76.5-96.7] |
| Tot ^a^ | 10122 | 11991 | 5330 | 2202 | 7921 | 4413 | 6460 | 10773 | 5986 | 5909 | 8584 |  |

The data are reported as absolute and (relative - %) abundance. The taxonomy was assigned to the sequence variants using the naive Bayesian classifier method implemented in DADA2 (18)^[[1]](#footnote-1)^. The Silva taxonomic training data formatted for DADA2 (version 132; 19) was used as a reference. In grey: taxa present in more than half (6/11) of the samples analyzed. ^a^ total number of reads after chimera removal (see **Table 1**). ^b^ expressed on the relative abundance values. NCS, negative control to 16S rRNA gene sequencing.

**Supplementary Figures**


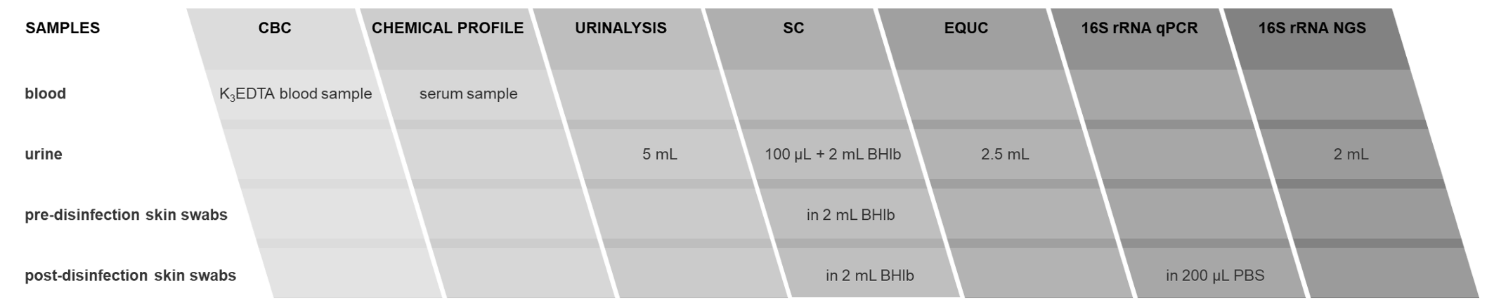


**Supplementary Figure 1.** Samples collected from each cat included in the study and analyses carried out. 16S rRNA, 16S ribosomal RNA; BHIb, brain heart infusion broth; CBC, complete blood count; EQUC, expanded quantitative urine culture; K3EDTA, K3 ethylene diamine tetra-acetic acid; NGS, next generation sequencing; PBS, phosphate-buffered saline; qPCR, quantitative real-time polymerase chain reaction; SC, standard culture.

1. DADA2 pipeline. <https://github.com/benjjneb/dada2> [Accessed February 01, 2020]. [↑](#footnote-ref-1)
